# Influence of the Amount of Change in Quadriceps Tendon Young’s Modulus on Amount of Change in Walking Speed before and after Total Knee Arthroplasty

**DOI:** 10.3390/medicina57121329

**Published:** 2021-12-04

**Authors:** Bungo Ebihara, Hirotaka Mutsuzaki, Takashi Fukaya, Koichi Iwai

**Affiliations:** 1 Graduate School of Health Sciences, Ibaraki Prefectural University of Health Sciences, 4669-2 Ami, Ami-machi, Inashiki-gun, Ibaraki 300-0394, Japan; 2 Department of Rehabilitation, Tsuchiura Kyodo General Hospital, 4-1-1 Otsuno, Tsuchiura, Ibaraki 300-0028, Japan; 3 Department of Orthopaedic Surgery, Ibaraki Prefectural University of Health Sciences, 4669-2 Ami, Ami-machi, Inashiki-gun, Ibaraki 300-0394, Japan; mutsuzaki@ipu.ac.jp; 4 Department of Physical Therapy, Faculty of Health Sciences, Tsukuba International University, 6-8-33 Manabe, Tsuchiura, Ibaraki 300-0051, Japan; t-fukaya@tius.ac.jp; 5 Center for Humanities and Sciences, Ibaraki Prefectural University of Health Sciences, 4669-2 Ami, Ami-machi, Inashiki-gun, Ibaraki 300-0394, Japan; iwai@ipu.ac.jp

**Keywords:** total knee arthroplasty, walking speed, quadriceps tendon, Young’s modulus, elastography, path analysis

## Abstract

*Background and Objectives*: Walking speed after total knee arthroplasty (TKA) is an important outcome. However, the effect of quadriceps tendon stiffness on walking speed remains unclear. This study aimed to clarify the influence of the amount of change in quadriceps tendon stiffness on the degree of change in walking speed before and after TKA. *Materials and Methods*: Sixteen patients who underwent TKA for knee osteoarthritis participated in this study (median age: 74.0 years (interquartile range: 64.5–75.8)). Shear-wave elastography was deployed to measure quadriceps tendon stiffness using Young’s modulus. A motion analysis system was used to assess kinematic parameters and walking speed. Participants’ knee circumference, range of motion, extension strength, one-leg standing time, walking pain level, and activity level were measured preoperatively and one year after TKA, and changes in values were calculated. We used path analysis to clarify the influence of the amount of change in the quadriceps tendon Young’s modulus on the change in walking speed. *Results*: The quadriceps tendon Young’s modulus negatively affected the knee flexion angle during swing (standardized partial regression coefficients (*β*) = −0.513, *p* = 0.042). The knee flexion angle during swing positively affected step length (*β* = 0.586, *p* = 0.017). Step length positively affected cadence (*β* = 0.733, *p* = 0.001). Step length and cadence positively affected walking speed *(β* = 0.563, *p* < 0.001, *β* = 0.502, *p* < 0.001, respectively). *Conclusions*: The amount of change in the quadriceps tendon Young’s modulus may affect the degree of change in walking speed after TKA through the amount of change in the knee flexion angle during swing, step length, and cadence. Clinically, reducing quadriceps tendon stiffness can be addressed in rehabilitation programs to increase walking speed after TKA.

## 1. Introduction

Knee osteoarthritis (OA) has a complex pathological mechanism that can induce cartilage destruction and inflammation, causing pain, stiffness, swelling, and loss of function [1]. Conservative treatments for knee OA include biomechanical interventions, intra-articular corticosteroids, exercise, self-management, education, strength training, weight management, and supplements for knee OA, including glucosamine sulphate, methylsulfonylmethane, and boswellic acids [2,3]. Walking speed is a risk factor for disability, cognitive impairment, institutionalization, falls, and/or mortality in older people [4]; it decreases as knee joint destruction progresses [5]. Total knee arthroplasty (TKA) is the gold-standard treatment for patients with severe knee OA [6]. Postural stability gradually recovers after TKA, and step speed, cadence, and semi-step length increase [7]. However, the decline in walking speed persists after TKA compared to that in healthy persons [8,9].

Factors related to walking speed include knee, hip, and ankle joint strength; knee joint range of motion (ROM); knee pain; and body mass index (BMI) [10,11,12,13,14]. Moreover, recent evidence has indicated, using structural equation modelling, that quadriceps tendon stiffness can affect walking speed through the knee flexion angle during swing, step length, and cadence in patients with knee OA [15]. However, it is not clear whether quadriceps tendon stiffness affects walking speed in TKA patients.

This study aimed to clarify whether the influence of the amount of change in quadriceps tendon stiffness affects the amount of change in walking speed before and after TKA according to the model used in a previous study [15]. Therefore, we hypothesized that the amount of change in quadriceps tendon stiffness from pre-operation to 1 year after TKA affects the amount of the change in walking speed. If this hypothesis is correct, improving quadriceps tendon stiffness can be clinically addressed in rehabilitation programs to increase walking speed after TKA.

## 2. Materials and Methods

### 2.1. Participants

This study was conducted between August 2018 and March 2021. The participants were patients with knee OA who underwent cruciate-retaining TKA using the medial para-patellar approach. Persona (Zimmer Biomet, Warsaw, IN, USA) was used as the artificial knee joint. Inclusion criteria included the ability to walk independently and no history of dementia or central nervous system disease. Exclusion criteria included onset of dementia and central nervous system disease or transfer to another hospital. Measurements were performed preoperatively and 1 year after TKA. The operated knees of the participants were evaluated for all measurements. In cases of bilaterally operated knees, all measurements were obtained from the knee with the lower preoperative maximum knee flexion angle during swing.

Preoperative measurements were obtained for 20 participants who met the inclusion criteria. However, two participants were transferred and one participant developed central nervous system disease. Moreover, one participant failed to return to the hospital due to anxiety related to COVID-19. Therefore, the 1-year postoperative measurements were performed for 16 participants.

We collected data on the participants’ age, sex, height, Kellgren–Lawrence grade [16], and femorotibial angle (FTA) from medical records. Body weight was measured using a digital scale and BMI was calculated.

### 2.2. Young’s Modulus of Quadriceps Tendon

Quadriceps tendon stiffness was measured using Young’s modulus [17] via an ultrasound Aixplorer ShearWave Elastography (SWE) system connected to a 2–10 MHz linear transducer (Supersonic Imaging, Aix-en-Provence, France). We selected the pre-set musculoskeletal and knee setting and the SWE Opt penetration mode. SWE was performed by the same physical therapist with two years of experience performing SWE measurements.

SWE was performed according to a procedure that showed good intra-rater reliability [18]. The Young’s modulus at 60° knee flexion can affect walking speed in patients with knee OA; therefore, measurements were performed with the participants in a supine position with 60° knee flexion [15]. Cushions were placed under the participants’ measurement knees and participants were instructed to remain relaxed. The linear transducer was then placed between the centre of the base of the patella and the muscle–tendon transition region of the rectus femoris muscle. Moreover, following a method that has been found to have good intra-rater repeatability [19], the linear transducer was placed just above the quadriceps tendon, 2 cm proximal to the bony insertion into the patella. Finally, a circular measurement range as large as possible within the quadriceps tendon was set. We recorded the mean values, as it is recommended to use mean values when using differently sized measurement ranges [20].

### 2.3. Knee Circumference

The knee circumference was measured using a tape measure. The participants were placed in a supine position. The participants’ measurement knees were extended as much as possible and relaxed. The circumference around the femur just above the base of the patella was measured and recorded. The measurements were performed so that the tape measure did not overlap with the patella.

### 2.4. Knee Joint ROM

Knee joint ROM was measured using goniometry. Knee flexion angles were measured through passive and active movements. First, the participants were placed in the supine position. The knees were then flexed as much as possible within the limit of the patient’s pain tolerance. Finally, we recorded the knee joint ROM from the intersection of the line connecting the greater trochanter of the femur and lateral epicondyle of the femur and the line connecting the fibular head and lateral malleolus of the fibula.

### 2.5. Knee Joint Extension Strength

Knee joint extension strength was measured using a Biodex System 3C dynamometer (Biodex Medical Systems, Shirly, NY, USA). We measured isometric knee extension torque at 70° knee flexion. First, participants sat on the seat of the Biodex System 3C dynamometer, in which the angle of the backrest was set to 85°. For the leg to be measured, we fixed the thigh and lower trunk to the seat and fixed the lower leg directly above the medial malleolus to the distal end of the dynamometer’s lever arm with straps. We instructed the participants to perform 5 s of maximal voluntary isometric contraction. Participants were given verbal encouragement to achieve maximal effort [21]. The measured peak knee extension torques were divided by each participant’s body weight [22]. We recorded peak knee extension torques and the knee extension torques/weight ratio.

Only one case was measured using a μTas F-1 (Anima, Tokyo, Japan) instead of the Biodex System 3C dynamometer. The reliability of intra-rater measurements using the μTas F-1 is good [23]. The participant sat on the bed with their knee bent at 70°. The μTas F-1 was fixed to the lower leg directly above the medial malleolus and the lower leg was fixed to the bed with a strap of the μTas F-1. The participant performed isometric contraction for 5 s with encouragement, and we measured the isometric knee extension force. Then, the knee extension torque was calculated by multiplying the measured knee extension force by the length from the lateral epicondyle of the femur to the part where the μTas F-1 was fixed. We recorded peak knee extension torques and the knee extension torques/weight ratio.

### 2.6. One-Leg Standing Time

The one-leg standing time was measured using a stopwatch. The procedure was based on previous research [24]. Participants stood on one leg with their hands at their sides and eyes open. We measured the time it took for the stance foot to shift, the hand to move away from the sides, or the lifted foot to touch the floor. The measurement was terminated if it exceeded 120 s. The measurements were performed twice, and the maximum values were recorded.

### 2.7. Walking Parameters

Walking parameters were measured using a motion analysis system with ten cameras (MA-3000) and four force plates (MG-1060) (Anima, Tokyo, Japan) at 100 Hz. We measured walking speed, step length, cadence, and maximum knee flexion angle during the swing phase. Reflective markers with a diameter of 20 mm were attached to the acromion, anterior superior iliac spine, greater trochanter, lateral epicondyle of the femur, lateral malleolus, and bone head of the fifth metatarsal bone. The data for the reflective marker position and ground reaction forces were low-pass filtered at 10 Hz and 20 Hz, respectively. The participants walked barefoot along a six-metre, level walkway at a normal walking speed. If the participants normally used a cane, they were permitted to use one during the trials. The software built into the MA-3000 calculated the average walking parameters of the four gait trials. These parameters were then recorded.

### 2.8. Walking Pain

Walking pain was measured using a visual analogue scale [25] immediately after the measurement of walking parameters. Participants marked their walking pain grades on a 100-mm linear scale. Then, each mark was read and converted to a score between 0 and 100 points. We recorded these values (no pain: 0 points; maximum pain: 100 points).

### 2.9. Activity Level

Activity level was measured by the knee scoring system of the Japanese Orthopaedic Association (JOA score), a confirmed, reliable tool [26]. There is a strong correlation between the activities of daily living grade of the Knee Injury and Osteoarthritis Outcome Score (KOOS) and the JOA score [27]. The JOA score consists of pain on walking, pain on ascending or descending stairs, range of motion, and joint effusion. The items are scored from 0 to 30 points, 25 points, 35 points, and 10 points, respectively. A high score indicates a good activity level. We recorded the total score (lowest: 0 points; highest: 100 points).

### 2.10. Statistical Analysis

We compared the measured values pre-operation and at a 1-year-postoperative visit. Moreover, we used a path analysis of the amount of change in the measurement values before and after TKA to analyse the relationship between the Young’s modulus and walking speed. The amount of change was calculated by subtracting the preoperative measurement value from the measurement value one year after TKA.

The distribution of the measured values and the amount of change were assessed using the Shapiro–Wilk test. Normally distributed values were calculated as means and standard deviation values; otherwise, median and interquartile range values were calculated.

Paired *t*-tests and Wilcoxon signed-rank tests were performed for comparison between the preoperative and 1-year-postoperative assessments. Specifically, paired *t*-tests were performed for the analysis of body weight, BMI, Young’s modulus, knee circumference, passive knee flexion angle, active knee flexion angle, knee extension torque, knee extension torque/weight ratio, step length, cadence, walking speed, and JOA score. Wilcoxon signed-rank tests were performed in the analysis of FTA, one-leg standing time, knee flexion angle during swing, and walking pain.

Path analyses [28,29] were performed to analyse the relationship between the amount of change in the Young’s modulus and walking speed. A previous study found that the passive knee flexion angle affected the Young’s modulus, the Young’s modulus affected the knee flexion angle during swing, the knee flexion angle during swing and the knee extension torque/weight ratio affected the step length, the knee flexion angle during swing and the step length affected the cadence, and the step length and cadence affected walking speed [15]. To confirm this relationship in patients with OA before and after TKA, we performed correlation and multiple regression analyses. Specifically, the correlation coefficients related to the amount of change in the passive knee flexion angle, knee extension torque/weight ratio, one-leg standing time, knee flexion angle during swing, cadence, and JOA score were determined using Spearman’s rank correlation coefficients. Otherwise, Pearson’s product-moment correlation coefficients were determined. Additionally, we performed multiple regression analyses. Following a model from a previous study, we performed a total of five multiple regression analyses with the amount of change in the Young’s modulus, knee flexion angle during swing, step length, cadence, and walking speed as independent variables. Finally, we created a path diagram related to the amount of change in walking speed based on the multiple regression analysis results.

*p*-values < 0.05 were considered statistically significant. The Shapiro–Wilk’s tests, paired *t*-tests, Wilcoxon signed-rank tests, correlation coefficient analyses, and multiple regression analyses related to the path analysis were performed using SPSS statistics version 26.0 (IBM Corp., Armonk, NY, USA).

## 3. Results

### 3.1. Physical Characteristics

The participants’ physical characteristics are summarized in Table 1. The median age of the participants was 74.0 years (interquartile range: 64.5–75.8).

### 3.2. Comparisons before and after TKA

Postoperative measurements were performed at a median of 364 (363–365) days after TKA. The comparisons before and after TKA are summarized in Table 2. There were significant differences for the FTA (*p* = 0.007), knee extension torque (*p* = 0.003), knee extension torque/weight ratio (*p* = 0.007), step length (*p* = 0.016), cadence (*p* = 0.007), walking speed (*p* = 0.006), walking pain (*p* = 0.002), and JOA score (*p* < 0.001) before and after TKA. There were no significant differences in other measurement values (*p* > 0.05).

### 3.3. The Amount of Change before and after TKA

The findings for the amount of change are summarized in Table 3. The amounts of change in the Young’s modulus and walking speed were −55.9 ± 137.4 kPa (mean ± standard deviation) and 0.16 ± 0.20 m/s, respectively.

### 3.4. Correlation Coefficient for Amounts of Change

The correlation analysis results for the amounts of change are summarized in Table 4. There was a negative correlation between the Young’s modulus and the knee flexion angle during swing (*p* = 0.015). There were positive correlations between the knee flexion angle during swing and step length (*p* = 0.049), cadence (*p* = 0.026), and JOA score (*p* = 0.005). There was a negative correlation between the knee flexion angle during swing and body weight (*p* = 0.022) and BMI (*p* = 0.015). There were positive correlations between step length and knee extension torque/weight ratio (*p* = 0.046), cadence (*p* = 0.002), and walking speed (*p* < 0.001). There were positive correlations between cadence and walking speed (*p* < 0.001). There were positive correlations between walking speed and knee extension torque/weight ratio (*p* = 0.025). There were no significant correlations in other measurement values (*p* > 0.05).

### 3.5. Path Analysis of the Amounts of Change

**Figure 1:**
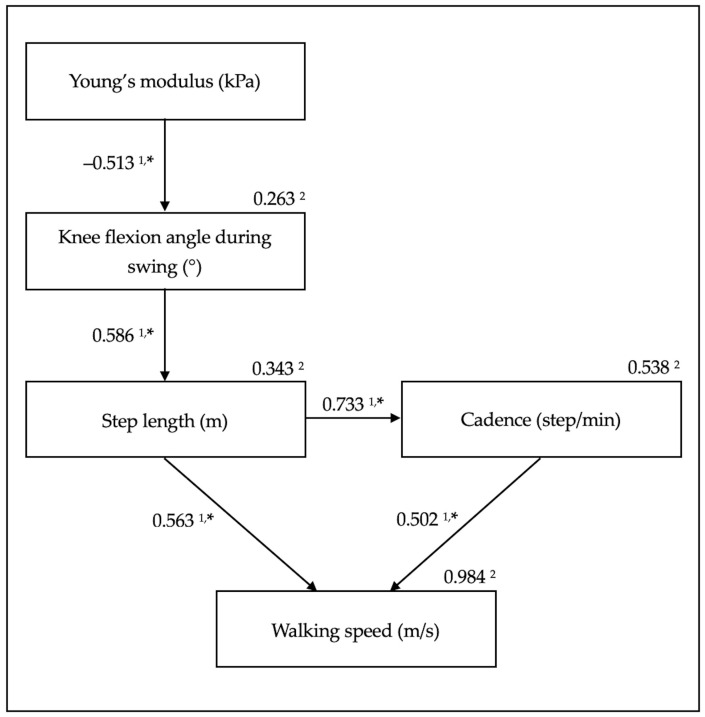
Figure showing path diagram for amounts of change. ^1^ Standardized partial regression coefficient. ^2^ Coefficient of determination. * *p* < 0.05.

The path analysis results for the amounts of change are summarized in Figure 1. The Young’s modulus negatively affected the knee flexion angle during swing (standardized partial regression coefficient (*β*) = −0.513, *p* = 0.042). The knee flexion angle during swing positively affected the step length (*β* = 0.586, *p* = 0.017). Step length positively affected cadence (*β* = 0.733, *p* = 0.001). Step length and cadence positively affected walking speed *(β* = 0.563, *p* < 0.001 and *β* = 0.502, *p* < 0.001, respectively).

## 4. Discussion

The path analysis for the amounts of change shown in Figure 1 supports our hypothesis that the amount of change in the quadriceps tendon Young’s modulus affects walking speed. The amount of change in the knee flexion angle during swing, step length, and cadence mediated these values.

Although the quadriceps tendon Young’s modulus did not appear to affect walking speed (there was no difference in the quadriceps tendon Young’s modulus before and after TKA), the effect size was 0.49. Therefore, focusing on the amount of change, path analysis revealed that the quadriceps tendon Young’s modulus did affect walking speed. The results of this study are similar to those of a previous report on the effect of the quadriceps tendon Young’s modulus on walking speed in patients with knee OA [15]. Quadriceps tendon stiffness can cause knee flexion limitation [30,31]. The quadriceps tendon Young’s modulus, which represents stiffness, can affect the knee flexion angle during swing [32]. Moreover, the knee flexion angle during swing can affect the speed of lower-limb swing, which can alter step length [12]. There is a relationship between step length and cadence, referred to as the walk ratio [33]. Therefore, when step length changes, the cadence can change, as can the walking speed. Based on the results of this study and the mechanism described above, when the amount of change in the quadriceps tendon Young’s modulus before and after TKA decreases—that is, when the quadriceps tendon becomes more flexible after TKA compared to before TKA—the knee flexion angle during swing may increase, step length and cadence may increase, and walking speed may increase.

The quadriceps tendon Young’s modulus at 60° knee flexion reflects the stiffness of the tendon and the flexibility of the quadriceps muscle and tendon. The stiffness of the quadriceps tendon and quadriceps muscle increases with knee flexion [34,35]. SWE confirms that the muscle–tendon unit elasticity decreases after static stretching and massage [36]. If stretching or massage improves the flexibility of the quadriceps muscle–tendon unit and reduces the quadriceps tendon tension at 60° of flexion, the Young’s modulus of knee flexion at 60° may decrease.

This study had four limitations. First, the number of participants was small. Performing structural equation modelling instead of path analysis on a larger sample would clarify the relationship between the quadriceps tendon Young’s modulus and walking speed. Second, the coefficients of determination of the amounts of change in the knee flexion angle during swing and the step length in the path diagram were relatively low. Therefore, they were insufficient to enable this path diagram to permit prediction of the amount of change in walking speed. The effect of the quadriceps tendon Young’s modulus on walking speed was partial, and stance-phase data would be needed to more accurately predict walking speed. Third, it is not known whether surgical techniques and artificial joint models other than those used in this study would affect the path diagram. Fourth, a μTas F-1 was used to measure knee extension strength in one case. This may have affected the outcomes for the knee extension strength. Further studies are required to address these limitations.

## 5. Conclusions

The amount of change in the Young’s modulus of the quadriceps tendon may affect the amount of change in walking speed after TKA through the amount of change in the knee flexion angle during swing, step length, and cadence. Our results indicate that reducing the Young’s modulus of the quadriceps tendon may increase walking speed one year after TKA. Clinically, reducing the stiffness of the quadriceps tendon can be addressed in rehabilitation programs for increasing walking speed after TKA.

## Figures and Tables

**Table 1:** Physical characteristics.

Characteristics	Pre-TKA (n = 16)
Age (years)	74.0 (64.5–75.8) ^2^
Sex (men/women)	3/13
Height (m)	1.52 ± 0.07 ^1^
Body weight (kg)	60.8 ± 11.4 ^1^
FTA (°)	180.0 (177.3–182.8) ^2^
KL grade (3/4)	4/12

^1^ Mean and standard deviation. ^2^ Median and interquartile range values. Abbreviations: pre-TKA, pre-total knee arthroplasty; FTA, femorotibial angle; KL, Kellgren-Lawrence.

**Table 2:** Comparison before and after TKA.

Measurement Item	Pre-TKA (n = 16)	Post-TKA (n = 16)	*p*-Value	Effect Size
Body weight (kg)	60.8 ± 11.4 ^1^	61.4 ± 12.1 ^1^	0.320 ^3^	0.05 ^5^
BMI (kg/m^2^)	26.2 ± 4.3 ^1^	26.5 ± 4.3 ^1^	0.368 ^3^	0.07 ^5^
FTA (°)	180.0 (177.3–182.8) ^2^	173.9 ± 3.7 ^1^	0.007 ^4,^*	0.68 ^6^
Young’s modulus (kPa)	306.0 ± 121.5 ^1^	250.1 ± 106.9 ^1^	0.125 ^3^	0.49 ^5^
Knee circumference (cm)	39.1 ± 3.3 ^1^	38.7 ± 3.5 ^1^	0.281 ^3^	0.12 ^5^
Passive knee flexion angle (°)	129.1 ± 12.6 ^1^	131.2 ± 11.7 ^1^	0.409 ^3^	0.17 ^5^
Active knee flexion angle (°)	121.8 ± 12.7 ^1^	120.9 ± 8.1 ^1^	0.761 ^3^	0.09 ^5^
Knee extension torque (Nm)	66.9 ± 20.0 ^1^	81.0 ± 18.6 ^1^	0.003 ^3,^*	0.73 ^5^
Knee extension torque/weight ratio (Nm/kg)	1.12 ± 0.34 ^1^	1.35 ± 0.34 ^1^	0.007 ^3,^*	0.68 ^5^
One-leg standing time (s)	9.1 (2.9–28.1) ^2^	8.0 (3.9–36.1) ^2^	0.301 ^4^	0.26 ^6^
Knee flexion angle during swing (°)	58.7 (51.9–64.3) ^2^	60.5 ± 5.0 ^1^	0.187 ^4^	0.33 ^6^
Step length (m)	0.49 ± 0.10 ^1^	0.53 ± 0.07 ^1^	0.016 ^3,^*	0.46 ^5^
Cadence (step/min)	110.3 ± 16.0 ^1^	121.5 ± 10.7 ^1^	0.007 ^3,^*	0.82 ^5^
Walking speed (m/s)	0.91 ± 0.26 ^1^	1.07 ± 0.17 ^1^	0.006 ^3,^*	0.73 ^5^
Walking pain (points)	29.9 ± 24.6 ^1^	0.0 (0.0–0.0) ^2^	0.002 ^4,^*	0.77 ^6^
JOA score (points)	64.1 ± 10.5 ^1^	85.0 ± 7.7 ^1^	<0.001 ^3,^*	2.27 ^5^

^1^ Mean and standard deviation. ^2^ Median and interquartile range values. ^3^ Paired *t*-test. ^4^ Wilcoxon signed-rank test. ^5^ Effect size d. ^6^ Effect size r. * *p* < 0.05. Abbreviations: pre-TKA, pre-total knee arthroplasty; post-TKA, post-total knee arthroplasty; BMI, body mass index; FTA, femorotibial angle; JOA, Japanese Orthopaedic Association.

**Table 3:** Degrees of change before and after TKA.

Measurement Item	Amount of Change (n = 16)
Body weight (kg)	0.6 ± 2.5 ^1^
BMI (kg/m^2^)	0.2 ± 1.0 ^1^
FTA (°)	−5.1 ± 5.9 ^1^
Young’s modulus (kPa)	−55.9 ± 137.4 ^1^
Knee circumference (cm)	−0.4 ± 1.5 ^1^
Passive knee flexion angle (°)	5.5 (−8.5–10.8) ^2^
Active knee flexion angle (°)	−0.8 ± 10.5 ^1^
Knee extension torque (Nm)	14.1 ± 16.3 ^1^
Knee extension torque/weight ratio (Nm/kg)	0.16 (−0.03–0.36) ^2^
One-leg standing time (s)	0.4 (−2.7–12.2) ^2^
Knee flexion angle during swing (°)	0.9 (−0.8–7.1) ^2^
Step length (m)	0.04 ± 0.06 ^1^
Cadence (step/min)	6.0 (0.9–18.8) ^2^
Walking speed (m/s)	0.16 ± 0.20 ^1^
Walking pain (points)	−28.6 ± 25.2 ^1^
JOA score (points)	20 (11.25–28.75) ^2^

^1^ Mean and standard deviation. ^2^ Median and interquartile range values. Abbreviations: BMI, body mass index; FTA, femorotibial angle; JOA, Japanese Orthopaedic Association.

**Table 4:** Correlation analysis results for amounts of change.

	Young’s Modulus	Knee Flexion Angle during Swing	Step Length	Cadence	Walking Speed
	Correlation coefficient				
Body weight (kg)	0.408 ^1^	−0.568 ^2,^*	−0.203 ^1^	−0.339 ^2^	−0.071 ^1^
BMI (kg/m^2^)	0.433 ^1^	−0.594 ^2,^*	−0.186 ^1^	−0.353 ^2^	−0.073 ^1^
FTA (°)	0.497 ^1^	−0.195 ^2^	−0.144 ^1^	−0.437 ^2^	−0.357 ^1^
Young’s modulus (kPa)	-	−0.594 ^2,^*	−0.196 ^1^	−0.465 ^2^	−0.285 ^1^
Knee circumference (cm)	0.230 ^1^	−0.439 ^2^	−0.425 ^1^	−0.222 ^2^	−0.210 ^1^
Passive knee flexion angle (°)	−0.124 ^2^	−0.103 ^2^	0.121 ^2^	0.310 ^2^	0.296 ^2^
Active knee flexion angle (°)	−0.085 ^1^	0.028 ^2^	0.161 ^1^	0.249 ^2^	0.211 ^1^
Knee extension torque (Nm)	0.263 ^1^	−0.085 ^2^	0.423 ^1^	0.344 ^2^	0.433 ^1^
Knee extension torque/weight ratio (Nm/kg)	0.091 ^2^	0.047 ^2^	0.504 ^2,^*	0.374 ^2^	0.557 ^2,^*
One-leg standing time (s)	0.368 ^2^	−0.274 ^2^	0.062 ^2^	−0.053 ^2^	0.071 ^2^
Knee flexion angle during swing (°)	−0.594 ^2,^*	-	0.499 ^2,^*	0.553 ^2,^*	0.457 ^2^
Step length (m)	−0.196 ^1^	0.499 ^2,^*	-	0.711 ^2,^*	0.931 ^1,^*
Cadence (step/min)	−0.465 ^2^	0.553 ^2,^*	0.711 ^2,^*	-	0.878 ^2,^*
Walking speed (m/s)	−0.285 ^1^	0.457 ^2^	0.931 ^1,^*	0.878 ^2,^*	-
Walking pain (points)	0.329 ^1^	−0.188 ^2^	−0.100 ^1^	−0.218 ^2^	−0.096 ^1^
JOA score (points)	−0.443 ^2^	0.659 ^2,^*	0.279 ^2^	0.327 ^2^	0.283 ^2^

^1^ Pearson’s product-moment correlation coefficient. ^2^ Spearman’s rank correlation coefficient. * *p* < 0.05. Abbreviations: BMI, body mass index; FTA, femorotibial angle; JOA, Japanese Orthopaedic Association.

## Data Availability

The data presented in this study are available on request from the corresponding author. The data are not publicly available due to privacy and ethical considerations.
